# Ion channels as convergence points in the pathology of pulmonary arterial hypertension

**DOI:** 10.1042/BST20210538

**Published:** 2021-08-04

**Authors:** Thibault R. H. Jouen-Tachoire, Stephen J. Tucker, Paolo Tammaro

**Affiliations:** 1 Clarendon Laboratory, Department of Physics, University of Oxford, Parks Road, Oxford OX1 3PU, U.K.; 2 Department of Pharmacology, University of Oxford, Mansfield Road, OX1 3QT Oxford, U.K.; 3 OXION Initiative in Ion Channels and Disease, University of Oxford, OX1 3PT Oxford, U.K.

**Keywords:** ion channels, patch clamp, potassium channels, pulmonary hypertension, vascular smooth muscle

## Abstract

Pulmonary arterial hypertension (PAH) is a fatal disease of the cardiopulmonary system that lacks curative treatments. The main pathological event in PAH is elevated vascular resistance in the pulmonary circulation, caused by abnormal vasoconstriction and vascular remodelling. Ion channels are key determinants of vascular smooth muscle tone and homeostasis, and four PAH channelopathies (*KCNK3*, *ABCC8*, *KCNA5*, *TRPC6*) have been identified so far. However, the contribution of ion channels in other forms of PAH, which account for the majority of PAH patients, has been less well characterised. Here we reason that a variety of triggers of PAH (e.g. *BMPR2* mutations, hypoxia, anorectic drugs) that impact channel function may contribute to the onset of the disease. We review the molecular mechanisms by which these ‘extrinsic’ factors converge on ion channels and provoke their dysregulation to promote the development of PAH. Ion channels of the pulmonary vasculature are therefore promising therapeutic targets because of the modulation they provide to both vasomotor tone and proliferation of arterial smooth muscle cells.

## Introduction

Pulmonary arterial hypertension (PAH) is a progressive disease of the cardiopulmonary system with life-limiting consequences. PAH is defined by a mean pulmonary artery pressure above 25 mmHg at rest, and end-expiratory pulmonary artery wedge pressure of less than 15 mmHg. Disease factors include: (i) genetic origins (i.e. mutations in genes crucial for pulmonary vascular function); (ii) other pathologies including congenital heart disease, portal hypertension, HIV or COPD; (iii) drugs or toxins, or (iv) unknown factors (i.e. PAH of idiopathic origins) [1]. In all cases, a complex pathological cascade leads to narrowing and remodelling of arteries and/or increased vascular reactivity ultimately leading to increased vascular resistance and elevated pulmonary blood pressure which may precipitate in right heart failure and death [1]. The development of PAH-specific therapies significantly improved patients’ outcome and quality of life [2]. However, poor prognosis (57% five-year survival rate), resistance to current drugs, and lack of curative treatment means there is an unmet need for new and innovative targets in PAH pharmacotherapy [2].

Ion channels have an important homeostatic function whereby they regulate the resting membrane potential and cytoplasmic [Ca^2+^] of pulmonary arterial smooth muscle cells (PASMC), which is a key determinant of vasoconstriction and vascular cell proliferation [3,4]. Therefore, the dual involvement of ion channels in the control of arterial tone and remodelling makes them potential key mediators in the pathogenic cascade of PAH, which can be therapeutically targeted. Supporting this view is the notion that mutations in ion channel genes may lead to hereditary forms of PAH [4,5]. Ion channel dysregulation may also be secondary to alterations in the cellular environment that take place during PAH, and thus contribute to worsening of the condition [6,7]. We therefore suggest that dysregulation of ion channels could be a convergence point in PAH pathogenesis, independent of the pathological trigger.

Here we examine the pathophysiological roles of ion channels in PAH, and how their dysfunction, either directly caused by genetic alterations in ion channel genes (‘intrinsic’ dysregulation) or secondary to altered cellular microenvironment (‘extrinsic’ factors), can lead to PAH. Since a range of recent reviews has covered the pathophysiology of PAH channelopathies [4,5], we primarily focus this review on the extrinsic factors (e.g. hypoxia, endothelial dysfunction, drugs, HIV) that contribute to dysfunction of plasmalemmal channels in PASMCs during PAH.

## Overview of channelopathies in PAH

A range of *de novo* and heritable mutations in PASMC ion channels have been implicated in familiar forms of PAH, these include mutations in the *KCNK3, ABCC8, KCNA5* and *TRPC6* genes. [4]

The TWIK-related acid-sensitive K^+^ (TASK) channels belong to the superfamily of Two-Pore Domain K^+^ (K2P) channels [8]. The TASK1 (*KCNK3*) channel is responsible for the background I_KN_ leak current that contributes to the resting membrane potential, vasomotor tone as well as proliferation of PASMCs [7,9]. So far, 12 loss-of-function missense mutations were identified in *KCNK3*, which was the first channelopathy discovered in PAH [5]. The lack of ‘protective’ heterodimerisation with TASK3 appears to underlie the lung-specific phenotype of *KCNK3* mutations [10]. Several *in vivo* studies confirmed that TASK1 loss-of-function precedes haemodynamic changes and is sufficient to cause PAH [7,9].

ATP-sensitive K^+^ (K_ATP_) channels promote hyperpolarisation and vasodilation in vascular smooth muscle cells [11]. Recently, 23 loss-of-function mutations in *ABCC8* (encoding the regulatory SUR1 subunit) were found in PAH patients [5,12]. These heterozygous mutations appear to impair channel trafficking and/or affect ATP sensitivity [13]. Several paradoxes surround this second PAH channelopathy, such as the lack of congenital hyperinsulinism, or the observation that patients with Cantú syndrome, with gain-of-function mutations in Kir6.1 and SUR2 subunits, also exhibit pulmonary hypertension [14]. The role of K_ATP_ in the pulmonary circulation therefore remains to be clarified.

In the pulmonary vasculature voltage-gated K^+^ (K_v_) channels also regulate the resting membrane potential, Ca^2+^ influx and vasoconstriction as well as cellular apoptosis, migration and proliferation [3,15]. Strong lines of evidence support a causal role for Kv1.5 dysfunction in PAH [16]. Sequencing of idiopathic PAH (IPAH) patients identified a number of mutations in the promoter and coding regions of *KCNA5* believed to affect its transcription, trafficking, and interaction with its β subunits [17,18]. A new variant has also been found in Pulmonary Hypertension of the Newborn (PPHN) [19]

Transient Receptor Potential Canonical (TRPC) channels are a subfamily of non-selective cation channels, which represent a major alternative route of Ca^2+^ entry outside voltage-gated Ca^2+^ channels, by functioning as both store-operated (SOC) and receptor-operated (ROC) channels [20]. Pharmacological blockade and knock-out experiments found a strong correlation between TRPC6 expression, capacitative Ca^2+^ entry, resting cytoplasmic [Ca^2+^], vascular tone and proliferation of rat PASMCs [21]. In PAH, increased TRPC6 expression promotes vasoconstriction and neomuscularisation of pulmonary arteries [22]. A genotypic analysis found that the 254(C → G) single nucleotide polymorphism (SNP) was 2.85 times more common in IPAH patients [23]. It is of interest that another mutation in *TRPC6* (F443I) was recently found to associate with PPHN [19].

## Extrinsic dysregulation of ion channels in PAH

**Figure 1.**
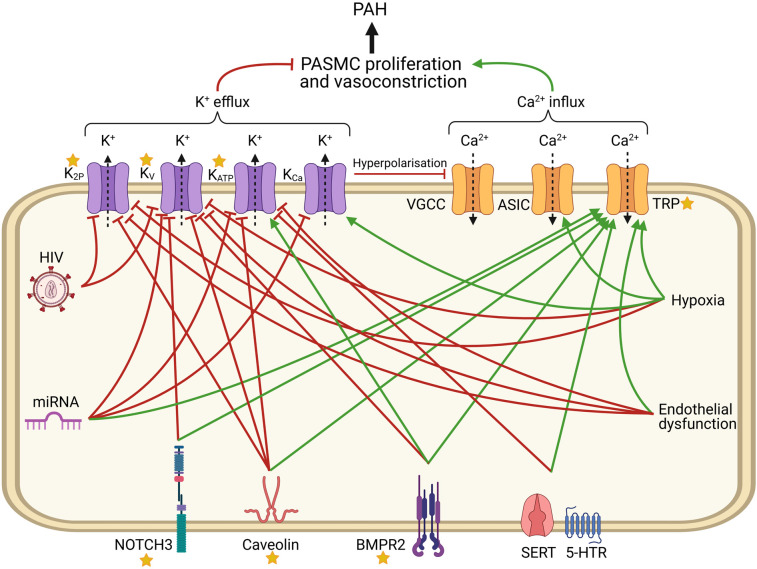
Dysregulation of PASMC ion channels during PAH. Red lines represent inhibitory influences vs activation for green arrows. Stars represent proteins with mutations associated with PAH.

Individuals with mutations in ion channels represent a minority of PAH patients, whereas dysregulation of ion channels is also observed in patients with other forms of PAH [6,7,24]. PAH can be induced by a wide variety of triggers, giving rise to different classifications (Table 1) [1]. In this section, the involvement of ion channels in the various forms of PAH is examined (Figure 1).

**Table 1:** Clinical classification of PAH

1. Pulmonary arterial hypertension (PAH)
1.1 Idiopathic PAH (IPAH)
1.2 Heritable/familial PAH (HPAH)
1.2.1 *BMPR2* mutations
1.2.2 Other mutations (e.g. *KCNK3, CAV1*)
1.3 Drug-induced PAH (DPAH) (e.g. fenfluramine)
1.4 PAH Associated with comorbidities (APAH)
1.4.1 Connective tissue disease (e.g. systemic sclerosis)
1.4.2 Human immunodeﬁciency virus (HIV) infection
1.4.3 Portal hypertension
1.4.4 Congenital heart disease
1.4.5 Schistosomiasis
1′. Pulmonary veno-occlusive disease and/or pulmonary capillary haemangiomatosis
1″. Persistent pulmonary hypertension of the newborn (PPHN)

### Germline mutations

#### BMPR2

Signalling events involving the transforming growth factor-β (TGFβ) family, in particular bone morphogenetic proteins (BMP), are an important regulator of PASMC homeostasis. More than 806 loss-of-function variants in bone morphogenetic protein receptor type II (BMPR2) have been identified, which account for 75% and 25% of heritable PAH (HPAH) and IPAH cases, respectively [24,25].

Several lines of evidence link BMP signalling to regulation of K^+^ channels in PASMC. Indeed, BMP2 up-regulates the expression of α subunits through Mothers Against Decapentaplegic Homologues (SMADs), while decreasing the expression of inhibitory β and γ subunits in human PASMC [26]. In a murine model of PAH, BMPR2 loss-of-function reduced Kv1.5 expression, and resulted in increased voltage-gated Ca^2+^ entry and vasoconstriction [27]. Additive effects between *BMPR2* and *KCNA5* mutations may underlie the earlier age of onset and higher disease severity in digenic patients, suggesting that mutational load and the presence of ‘second hits’ is important in PAH progression [28]. *BMPR2* deletion was also shown to down-regulate TASK1 and promote vasoconstriction in rat PASMC, although in this case modulation appears to be transcription-independent, but instead due to altered channel trafficking [25]. *ABCC8* mRNA expression was also up-regulated in lung biopsies obtained from BMPR2 patients [29].

**Figure 2.**
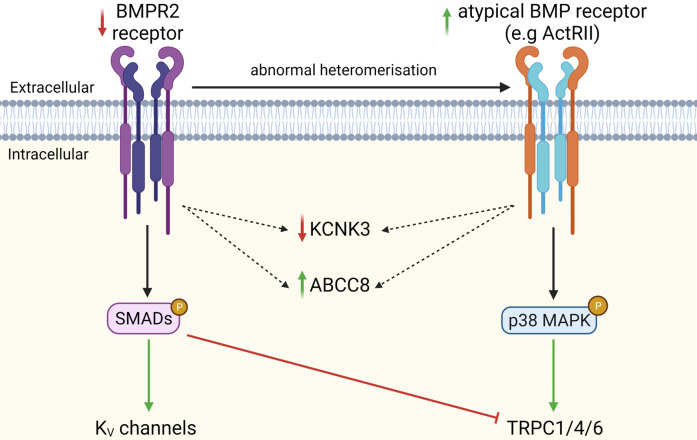
Consequences of shift from canonical BMPR2 to non-canonical BMP signalling on ion channel function in PASMCs. Continuous arrows denote inhibition/down-regulation (red) or activation/up-regulation (green). Dotted arrows represent undefined links. ‘P’ denotes phosphorylated, activated mediator. MAPK, mitogen-activated protein kinase; ActRII, activin type 2 receptor.

BMPR2 mutations also promote the formation of atypical heteromeric BMP receptors, shifting BMP signalling towards non-canonical MAPK-dependent pathways in murine PASMCs [30,31]. This shift is important, as BMP2 was shown to inhibit the expression of TRPC1/4/6 channels and proliferation of proximal rat PASMCs, whereas BMP4 up-regulated TRPC1/4/6 expression and distal PASMCs proliferation via non-canonical cascade PASMCs [32,33]. Overall, BMPR2 haploinsufficiency favours the formation of different BMP receptor complexes, suppressing the canonical, pro-apoptotic BMP2/SMAD signalling in proximal PASMCs, and potentiating the non-canonical, pro-proliferative BMP4/p38 axis in distal human PASMCs (Figure 2) [34,35].

#### Caveolin

The scaffolding protein caveolin is a structural component of caveolae, which are specialised dynamic lipid rafts important for microdomain functional coupling [36]. For instance, in the pulmonary vasculature, the caveolin ‘signalplex’ regulates arterial vasoconstriction by interacting with Ca^2+^ regulatory molecules [36]. Several mutations that decrease caveolin-1 (*CAV1*) expression were found in PAH, and *CAV1* knock-out in animal models leads to pulmonary hypertension [37,38]. Contrary to the loss of expression in pulmonary endothelium, CAV1 expression is up-regulated in rat PASMCs and promotes proliferation [39]. Caveolin-1 directly influences the kinetics, assembly and regulation of several vascular channels via its scaffolding domain. For instance, binding of caveolins to Kir6.1, Kv1.5 and TASK1 reduced their functional activity in heterologous expression systems [40–42]. Caveolin-1 is also a positive regulator of TRPC assembly and SOCE in mice pulmonary vascular cells [43]. Increased caveolin expression in PASMCs of two rat models of PAH (chronic hypoxia and monocrotaline) was found to enhance agonist-induced SOCE/ROCE and vasoconstriction in pulmonary arteries [44].

#### NOTCH3

The transmembrane NOTCH3 receptor controls the differentiation of PASMCs from proliferative to contractile phenotype [45]. Three genetic variants in *NOTCH3* were linked to PAH [19,46]. Clinical studies found a causative role for the overexpression of NOTCH3 in the abnormal proliferation of PASMCs in PAH [47,48]. NOTCH3 enhanced Ca^2+^ entry in human PASMCs by (i) up-regulating TRPC6-dependent SOCE and (ii) inhibiting Kv1.2/Kv1.5 activity, which in turn promotes voltage-gated Ca^2+^ entry [45,49]. The resulting increased Ca^2+^ mobilisation leads to PAH by preferentially promoting vascular remodelling [47]. The fact that some of these effects were transcription-independent hints that non-canonical signalling of NOTCH3 may be involved in this pathological process [45,49]. Indeed, the two NOTCH3 missense mutations were shown to boost proliferative signalling of NOTCH3, despite actually decreasing its downstream transcriptional activity [46]. The NOTCH intracellular domain (NICD) has also been proposed to directly influence ion channel activity [45,49].

### Epigenetics

MicroRNAs (miRNAs) are small non-coding RNAs that regulate gene expression by binding and silencing the translation of target mRNAs. Several studies have shown a correlation between dysregulation of miRNAs, altered ionic currents and dysfunction of rat and human pulmonary artery *in vivo*. For example, the Kv1.2, Kv1.5, Kv7.5, BKCaβ1 and TASK1 channels are under direct negative control of miR-1, miR-23b-3p, miR-29b, miR-138, miR-190, miR-206 and miR-222, whose up-regulation in PAH (e.g. 4-fold for miR-1) decreased channel expression in human and rodent PASMCs, and promoted membrane depolarisation and arterial wall hypertrophy [50–52]. In addition, down-regulation of miR-135a-5p increased TRPC1 expression and promoted PASMC proliferation [53]. miRNAs are promising diagnostic markers of PAH, as well as potential therapeutic targets to correct ion channel dysfunction in PAH.

### Comorbidities and PAH

#### HIV

PAH is one of the most severe complications of HIV infection and a major cause of mortality in HIV-PAH patients, leading to a lower survival rate compared with other PAH patients [54]. HIV infection increases the incidence of PAH by 2500-fold in human subjects [54], and can trigger PAH in some laboratory animals (rats and macaques, but not mice) [55–57]. Two studies showed that HIV transgenes down-regulated the I_KN_ and I_KV_ currents (decreased Kv1.5, Kv7.1 and Kv7.4 expression) in rodent pulmonary arteries, possibly contributing to membrane depolarisation [55,57]. The Vpu protein of HIV-1 shares a high homology with the N-terminal of TASK1 channel, and it promotes abnormal oligomerisation of Vpu and TASK1 subunits, which leads to their degradation in heterologous expression systems [58]. The connection between HIV and PAH remains to be determined, one question being why only a small proportion (8.3%) of HIV patients have PAH.

#### Hypoxia

Hypoxia is a known trigger of pulmonary hypertension and ion channels are essential mediators of the hypoxic pulmonary vasoconstriction (HPV) [59]. The inhibition of Kv1.5 and Kv2.1 channels in the small resistance pulmonary arteries is a major contributor of HPV [15,60], but the underlying regulatory mechanisms remain controversial [61,62]. Although redox signalling is a key component of HPV, the extent of changes in the level of reactive oxygen species (ROS) during hypoxia are not fully defined, and both a decrease and an increase in ROS were shown to inhibit Kv channel opening [61]. The molecular mechanisms of oxygen sensing in PASMCs are also not fully defined. Mitochondria, which produce ROS in proportion to PO_2_ levels, are likely involved in the PASMC response to hypoxia [62]. Cytoplasmic NADPH oxidases, which also produce ROS, reportedly lead to inhibition of Kv1.5 channel activity and modulation of channel trafficking via oxidation of C-terminal cysteine residues [63]. Also the TASK1 channel mediates an O_2_-sensitive current in carotid body cells [64]. Conversely, hypoxia down-regulated both TASK1 expression (via the RELMβ-STAT3-NFAT pathway) and activity (via the kinase Src), which promote human PASMC proliferation and vasoconstriction [65,66].

Hypoxia led to activation of store-operated Ca^2+^ entry (SOCE) and elevation of cytoplasmic [Ca^2+^] in rodent PASMCs [67]. Mice in which the *Trpc1* and *Trpc6* genes were deleted (knockouts) had impaired hypoxic response and loss of hypoxic vasoconstriction [22]. The rise in ROS up-regulates both the expression and assembly of SOCE channels to promote Ca^2+^ mobilisation in hypoxic PASMCs [68,69]. There is evidence indicating that hypoxia directly modulates TRPC6 via the second messenger diacylglycerol and the enzyme AMP-activated protein kinase. Calcium Release-Activated Calcium (ORAI) channels are involved in the control of proliferation of rat PASMCs, but their role in PAH requires further investigation [21].

The mechanisms of chronic hypoxia, more relevant to the context of PAH, may differ from those of HPV. Hypoxia-inducible factors (HIFs) are key transcription factors that contribute to remodelling of pulmonary artery during chronic hypoxia [70]. Selective down-regulation of the expression of Kv α subunits (Kv1.1, Kv1.5, Kv2.1, Kv4.3, Kv9.3) in PASMC via HIF1α occurs during chronic hypoxia; this signalling pathway is abnormally potentiated by ROS during PAH [71,72]. In contrast, hypoxia increased the expression of Ca^2+^-activated K^+^ (BKCa) channels in rat PASMCs, which may explain the BKCa up-regulation seen in pulmonary arteries of PAH patients [73]. The underlying mechanism involves altered expression of the auxiliary β1 subunit via HIF1α, which enhances BKCa channel sensitivity to voltage and Ca^2+^, hence promoting PASMC relaxation and thus constituting an endogenous protective mechanism [74].

Chronic hypoxia also selectively up-regulates a range of non-selective cation channels in PASMCs including TRPC1 and TRPC6, via the HIF1α and Notch signalling pathways, and the osmo-mechanosensitive cation channels, TRPV1/4 [75–77]. The family of acid-sensitive ion channels (ASIC) can also participate in SOCE in pulmonary vascular cells [67]. ASIC1-dependent Ca^2+^ entry during hypoxia is independent of changes in gene expression [67], but it depends on inactivation of the inhibitory effect of H_2_O_2_ on ASIC1 channel, as well as stimulation of forward trafficking via the protein RhoA [67,78]. The increase in cytoplasmic [Ca^2+^] in PASMCs observed in hypoxia led to activation of transcription factor NFAT which down-regulates Kv1.5 expression and mitochondrial homeostasis, potentially hardwiring ionic dysfunction in a pathological feedback loop [79].

### 5-HT Signalling and anorectic drugs

Appetite-suppressants that potentiate serotonin signalling (e.g. fenfluramine, benfluorex) were taken off market after causing a drug-induced PAH epidemic in the 1960–70s [80]. The causal role of up-regulated 5-HT signalling in PASMCs of PAH patients and animal models further reinforced this ‘serotonin hypothesis’ of PAH [81]. The serotonin transporter (SERT) is strongly implicated in the pathogenesis of PAH [82], and some studies have linked a gain-of-function polymorphism in SERT to an increased risk of PAH [83].

5-HT is both a potent vasoconstrictor and PASMC mitogen, known to up-regulate TRPC1, TRPC6, TRPV4 currents in rat PASMCs [84,85]. Anorectic drugs constrict pulmonary arteries by inhibiting Kv1.5 currents, although the underlying molecular mechanisms are not fully understood and may include direct K_V_ channel inhibition, activation of 5-HT_2_ receptors or internalisation by SERT [86]. The pathways downstream of the 5-HT receptor and transporter seem to converge on second messengers like ROS [87] and kinases [86], which are known modulators of ion channel activity. For instance, 5-HT triggers the endocytosis of Kv1.5 channels in PASMC through a 5-HT_2A_R/tyrosine kinase/caveolin pathway [88].

### Endothelial dysfunction

The endothelium produces several vasoactive mediators that play an important role in PASMC homeostasis. A shift towards vasoconstrictive and mitogenic endothelial signals is one of the first events in PAH. It is still unclear whether endothelial dysfunction is a cause or consequence of PAH; however a monocrotaline-induced animal model of PAH presents pulmonary endothelium dysfunction, which is targeted by current PAH therapies (e.g. endothelin receptor antagonists, prostacyclin analogues) [89]. Endothelial dysfunction is accompanied by up-regulation of endothelin-1 and down-regulation of NO and PGI_2_ signalling [89]. These factors modulate vascular ion channels through cyclic nucleotides and protein kinases [90]. Endothelin-1 promotes depolarisation in rodent and human PASMCs and vasoconstriction by inhibiting I_KV_, I_KATP_ and up-regulating I_CaCl_, I_BKCa_ and TRPC currents through the PLC/PKC pathway [90]. Endothelin-1 also inhibits TASK1 in PASMC, which could involve both gating inhibition in TASK1 as well as its phosphorylation-dependent internalisation [91,92]. The NO/cGMP/PKG pathway promotes vasorelaxation by activating I_Kv_, I_KCa_, I_KN_, I_KATP_, and inhibiting I_SOCE_ [90,93]. Likewise, the PGI_2_/ cAMP/PKA axis activates I_Kv_, I_KCa_, I_KN_, I_KATP_ and inhibits TRPC channels [90,94].

## Summary and implications for therapy

Ion channels are key determinants of the tone and the remodelling of the pulmonary artery. Over 50 genetic variants in four ion channels (*KCNK3*, *ABCC8*, *KCNA5* and *TRPC6*) have been found in PAH patients [4]. In this review, we show that ion channel dysfunction occurs in virtually in all forms of PAH (Figure 1).

A better characterisation of the regulation of ion channels in PAMSCs could provide new information about why so many different triggers (e.g. anorectic drugs, HIV, hypoxia) can all lead to PAH. Integrative ‘omic’ approaches hold hope for establishing a deep genotype-phenotype correlation of the disease. Identifying new pathways that regulate PASMCs ion channels will be important to draw an accurate picture of their pathological relevance in the various forms of PAH (e.g. the recently proposed link between TASK1 and vitamin D deficiency) [95]. Also, the molecular mechanisms that dysregulate the Kv7 channel [96], the mechanosensitive Piezo1 channel [97] and the Ca^2+^-activated Cl^−^ channel TMEM16A [98] in PAH remain poorly understood.

Multiple studies showed that activators of K^+^ channels [7,99] and inhibitors of both Cl^-^ channels [98] and TRPC channels [100] can prevent and/or reverse PAH in animal models. Optimising the selectivity, potency and lung-specific delivery of these compounds will therefore be paramount to the development of new PAH therapies. Recent advances in the determination of structures for these channel types may also expedite drug discovery efforts towards this goal

## Perspectives

- **Highlight the importance of the field:** Pulmonary arterial hypertension is a multifactorial and severe disease with no curative treatments. Vasoconstriction and arterial remodelling both contributes to elevated resistance to blood flow and increased blood pressure.
- **A summary of the current thinking:** Ion channels are involved in the control of both arterial tone and proliferation of pulmonary artery smooth muscle cells. Dysfunction of ion channels plays a causative role in PAH, and may link the different pathological triggers of PAH to the onset of the disease.
- **A comment on future directions:** Ion channels are currently under-explored targets for PAH. Understanding the regulation and pathophysiological role of ion channels in PAH will provide new avenues for therapeutic intervention.

## Open Access Statement

Open access for this article was enabled by the participation of University of Oxford in an all-inclusive Read & Publish pilot with Portland Press and the Biochemical Society under a transformative agreement with JISC.

## Abbreviations

BMP: bone morphogenetic proteins
BMPR2: bone morphogenetic protein receptor type II
CAV1: caveolin-1
HIF: hypoxia-inducible factor
HPAH: heritable pulmonary arterial hypertension
HPV: hypoxic pulmonary vasoconstriction
IPAH: idiopathic pulmonary arterial hypertension
K_ATP_: ATP-sensitive K^+^
PAH: Pulmonary arterial hypertension
PASMC: pulmonary arterial smooth muscle cells
PPHN: Pulmonary Hypertension of the Newborn
ROS: reactive oxygen species
SERT: serotonin transporter
SOCE: store-operated Ca^2+^ entry
TASK1: TWIK-Related Acid Sensitive K^+^ Channel 1
TRPC: Transient Receptor Potential Canonical
